# Epoxy-Based Vitrimers for Sustainable Infrastructure: Emphasizing Recycling and Self-Healing Properties

**DOI:** 10.3390/polym17030373

**Published:** 2025-01-30

**Authors:** Myung Kue Lee, Min Ook Kim, Taehwi Lee, Sanghwan Cho, Dongchan Kim, Wonghil Chang, Yongseok Kwon, Seongkwan Mark Lee, Ju Kwang Kim, Bong Cheol Son

**Affiliations:** 1 Department of Civil and Environmental Engineering, Jeonju University, 303 Cheonjam-ro, Wansan-gu, Jeonju-si 55069, Jeollabuk-do, Republic of Korea; concrete@jj.ac.kr (M.K.L.); wchang@jj.ac.kr (W.C.); jisankys@jj.ac.kr (Y.K.); 2 Department of Civil Engineering, Seoul National University of Science and Technology, 232 Gongneung-ro, Nowon-gu, Seoul 01811, Republic of Korea; lth20910@seoultech.ac.kr (T.L.); sanghc9812@naver.com (S.C.); kdc0357@seoultech.ac.kr (D.K.); 3 School of Liberal Studies, Kunsan National University, 558 Daehak-ro, Gunsan-si 54150, Jeollabuk-do, Republic of Korea; marklee@kunsan.ac.kr; 4 IAN GEOTEC, 39 Nangsan Agricultural Complex-gil, Nangsan-myeon, Iksan-si 54521, Jeollabuk-do, Republic of Korea; geoskorea@naver.com; 5 GROVES, 102-19, Sinbok-ro, Deokjin-gu, Jeonju-si 54842, Jeollabuk-do, Republic of Korea; grovesboss@naver.com

**Keywords:** epoxy-based vitrimers, recyclability, self-healing, sustainable infrastructure, civil engineering applications

## Abstract

Epoxy-based vitrimers represent a paradigm shift in material science, offering an unprecedented combination of mechanical robustness, environmental sustainability, and reconfigurability. These dynamic polymer systems utilize associative dynamic covalent bonds (DCBs) such as transesterification to blend the structural integrity of thermosets with the recyclability and self-healing properties of thermoplastics. This unique combination makes vitrimers ideal candidates for high-performance applications in industries such as civil engineering, where material durability, repairability, and environmental compatibility are critical. Epoxy-based vitrimers, in particular, exhibit exceptional self-healing capabilities, allowing them to autonomously repair microcracks and damage, restoring mechanical properties under appropriate stimuli such as heat or light. Their recyclability further aligns with global sustainability goals by reducing material waste and lifecycle costs. Recent advancements have also integrated bio-based feedstocks and scalable manufacturing methods, enhancing the feasibility of these materials for industrial applications. This review explores the underlying self-healing mechanisms, dynamic recycling processes, and the emerging role of epoxy-based vitrimers in civil engineering. Challenges related to scalability, mechanical optimization, and regulatory acceptance are also discussed, with a focus on their potential to drive sustainable innovation in infrastructure materials.

## 1. Introduction

The development of advanced materials has long been a cornerstone of addressing the growing demands of modern society. Among these innovations, vitrimers stand out as a groundbreaking advancement in material science, offering a unique combination of mechanical robustness, environmental sustainability, and reconfigurability [1,2,3,4]. These dynamic polymer systems utilize the principles of covalent adaptable networks (CANs) to combine the mechanical stability of thermosets with the recyclability and self-healing capabilities traditionally associated with thermoplastics [5,6,7]. By leveraging associative dynamic covalent bonds (DCBs), such as transesterification, vitrimers exhibit remarkable thermal adaptability and mechanical durability, making them suitable for high-performance industries such as aerospace, automotive, and civil infrastructure [8,9,10].

**Figure 1:**
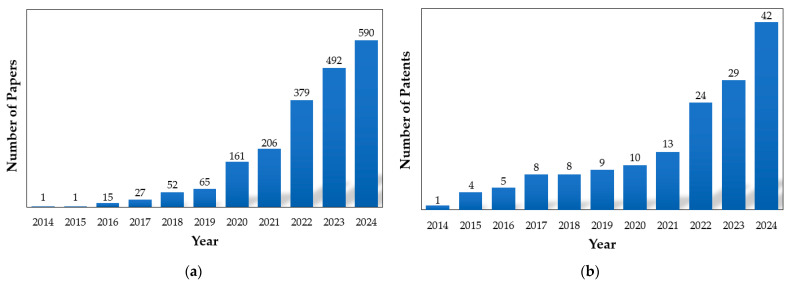
Number of published (**a**) papers and cumulative (**b**) patents from 2014 to 2024, identified through Google Scholar using the search term ‘Epoxy Vitrimers’ in the article title.

Epoxy vitrimers are particularly notable for their structural advantages and ease of functionalization [9,11,12]. The incorporation of DCBs into the epoxy network allows these materials to maintain their crosslinked structure under operational conditions while enabling topological rearrangements at elevated temperatures [12]. Previous studies have demonstrated their exceptional recyclability and mechanical resilience, even under extreme thermal and chemical conditions [13,14]. For example, dynamic crosslinking mechanisms enhance stress relaxation and adaptability [15,16]. Additionally, significant progress has been made toward industrial scalability through solvent-free, high-shear reactive mixing techniques that improve both environmental sustainability and economic feasibility [17,18]. The integration of renewable bio-based components, such as lignin and rosin derivatives, further addresses environmental concerns associated with traditional epoxy systems [19]. The growing interest in epoxy vitrimers is evidenced by a consistent increase in publications within the global scientific and patent literature over time (Figure 1).

**Figure 2:**
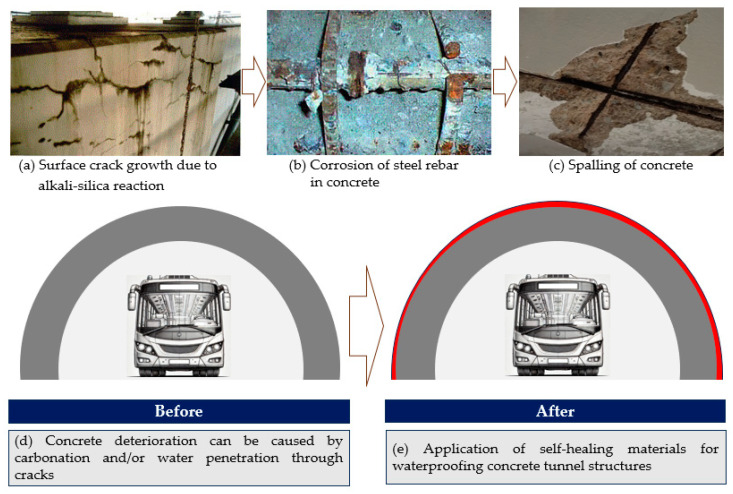
Schematic representation showing the application of self-healing materials to concrete tunnel structures for waterproofing purpose.

In civil engineering, materials must show outstanding durability, cost-efficiency, and environmental sustainability [20,21,22,23]. These qualities are critical for promoting sustainable construction, reducing energy consumption and CO_2_ emissions, and ensuring long-term structural performance throughout a structure’s lifecycle [24]. Traditional thermosets, despite their structural robustness, fall short of modern sustainability goals due to their irreversible and non-recyclable nature [25]. Epoxy-based vitrimers, with their dynamic covalent frameworks, bridge this gap by offering reprocessability and self-healing capabilities [3,5]. These features not only lower the lifecycle costs of infrastructure projects but also reduce environmental impact by minimizing material waste. Recent advancements have highlighted the utility of vitrimers in high-stress applications, such as coatings and adhesives, where their self-healing properties significantly enhance service life and reliability [26,27]. Moreover, their tunable thermal and mechanical properties make them ideal for use in harsh environmental conditions [28]. The advantages of epoxy-based vitrimers extend beyond their material properties. Their development aligns with global efforts to promote a circular economy and reduce plastic waste. By enabling recyclability without compromising structural integrity, these materials address critical environmental challenges [17]. Their compatibility with bio-based feedstocks also opens new opportunities for sustainable manufacturing practices [10,19]. The versatility of these systems—demonstrated through shape-memory effects, stress relaxation properties, and resistance to thermal degradation—positions them as frontrunners in next-generation material solutions [4,29,30,31]. Thus, it can be said that integrating epoxy-based vitrimers into civil infrastructure allows industries to achieve a sustainable balance between performance and environmental responsibility. Figure 2 shows a schematic representation showing the application of self-healing materials to old concrete structures for waterproofing purposes. Figure 2a–c illustrate common forms of deterioration observed in aging concrete structures, including tunnels. In contrast, Figure 2d,e demonstrate the protective effects of self-healing materials in safeguarding tunnel structures against harmful ions and water infiltration.

Vitrimers are known for their exceptional recycling and self-healing abilities, enabled by DCBs. These bonds allow the polymer network to break and reform, making the material remoldable and repairable. Typically, these dynamic bonds form through reversible reactions like transesterification or thiolene click chemistry, which can be triggered by external stimuli [32,33]. When broken, the polymer can be reshaped or repaired, and once reformed, the material regains its original properties. These unique recycling and self-healing properties make vitrimers suitable for various applications, such as recyclable plastics and self-healing adhesives. For example, a boronic ester vitrimer was synthesized from renewable diols derived from eugenol and diphenyl boronic acid [34]. Despite being highly crosslinked, the crosslinking bonds remain dynamic, allowing the vitrimer to be reshaped or repaired when heated to 150 °C and pressure is applied. This eugenol-based boronic ester vitrimer is a sustainable material option due to its recyclability and self-healing features, making it ideal for applications that demand durability [35]. In some cases, vitrimers exhibit intrinsic self-healing that occurs repeatedly without external triggers. For instance, poly(propylene oxide)-based benzoxazines with acid-functionalized benzoxazines demonstrate self-healing behavior when incorporated into a polydimethylsiloxane matrix, driven by metal-ligand interactions and supramolecular attractions. This versatility positions vitrimers as promising materials for innovation in sustainable and durable biomaterials. The fundamental chemistry lies in the formation of dynamic covalent bonds–in this case, ester linkages capable of undergoing exchange through transesterification reactions:

R–COOR’ + R’’–OH ⟶ R–COOR’’ + R’–OH

This equilibrium is readily shifted by altering factors such as temperature, catalyst type, and network composition. The result is a polymer matrix that behaves as a solid under ambient conditions yet can be “rearranged” or “flow” when transesterification is activated. Epoxy-based vitrimers are generally synthesized by reacting an epoxy resin with a carboxylic acid-functional curing agent, thereby generating ester bonds and hydroxyl (–OH) groups along the polymer backbone. A common example involves diglycidyl ether of bisphenol A (DGEBA) and citric acid, as illustrated:

Epoxy (R–CH–CH_2_–O) + Carboxylic Acid (R’–COOH) ⟶ Ester Bond (R–COO–CH–CH_2_–R’) + Hydroxyl (–OH)

This initial reaction not only cross-links the network but also introduces hydroxyl groups, which are critical for subsequent transesterification. The hallmark of a vitrimer’s dynamic nature is the ability of hydroxyl groups in the network to act as nucleophiles. Under appropriate conditions (e.g., elevated temperature, presence of a catalyst), a hydroxyl group (R′′–OH) attacks the carbonyl carbon of an existing ester (R–COOR′), forming a tetrahedral intermediate. This intermediate then collapses, releasing the original alcohol (R′–OH) and creating a new ester (R−COOR′′). The overall process is reversible and can be repeated multiple times. A variety of metal-based catalysts (e.g., zinc acetate, tin(II) 2-ethylhexanoate) or organobases can lower the activation energy for transesterification. By tuning catalyst concentration and selection, researchers can modulate reaction kinetics, enabling control over the speed of bond exchange, the temperature at which flow occurs, and the degree of healing or recycling efficiency achieved.

While the last five years have seen a number of comprehensive reviews on vitrimers’ underlying chemistry, mechanical properties, and potential for various advanced applications, most of these publications have not focused on, or thoroughly explored, the specific challenges and requirements associated with civil infrastructure [36,37,38,39]. For foundational insights into vitrimer design, synthesis, and emerging applications in fields such as electronics, automotive, or biomedical devices, readers may wish to consult. The present work serves a distinct purpose by providing a focused analysis of the remaining hurdles to be overcome before vitrimers can transition into large-scale infrastructure projects (e.g., bridges, highways, and buildings). Specifically, we address technical barriers such as cost-effective scaling, long-term durability, and compatibility with existing codes and standards—topics often only briefly addressed, if at all, in broader vitrimer reviews. By clarifying these practical limitations and spotlighting current research needs, our goal is to offer a roadmap for researchers, engineers, and industry stakeholders seeking to adapt vitrimers for infrastructure applications, without duplicating the comprehensive overviews already available in the literature. Numerous experimental studies have previously explored the potential applications of vitrimers across various fields, including aerospace and mechanical engineering, as summarized earlier. However, research on the use of epoxy vitrimers in civil engineering remains limited. This review consolidates recent advancements in epoxy-based vitrimers, focusing specifically on their dynamic recycling mechanisms, self-healing capabilities, and potential applications in civil infrastructure. It highlights the transformative potential of vitrimers to promote sustainable development and drive innovative engineering solutions.

## 2. Polymer-Modified Cementitious Materials for Concrete Repair

The success of concrete repairs largely depends on the properties of repair materials itself and also the interfacial bond between the applied material and the existing concrete substrate [40]. Extensive experimental research has been conducted to clarify the influence of various polymer types on the mechanical properties and durability of polymer-modified cementitious composites (PCCs) [41,42,43,44,45,46,47,48,49,50,51,52,53,54,55,56,57].

Among these, epoxy-modified cementitious composites (ECCs) have garnered significant attention due to their excellent compatibility with cement-based materials [44]. For instance, studies on water-based epoxy resin-modified cement mortar for repairing deteriorated concrete exposed to high temperatures revealed that ECCs exhibit strong adhesion under thermal stress caused by differing thermal expansion coefficients [45]. Research also indicates that incorporating aqueous epoxy resin into Portland cement improves flexural strength. Specifically, a 5% epoxy resin content by weight of cement yielded the highest mechanical and interfacial bonding strength [46]. However, exceeding 10% resin content resulted in reduced mechanical properties, likely due to residual, unhardened epoxy resin hindering hydration and polymerization processes [47,48]. The performance of other polymers, such as styrene-butadiene rubber (SBR), has also been studied and recommended to maintain SBR replacement ratios below 5% for optimal performance [49,50]. Furthermore, excessive epoxy replacement ratios (above 10%) negatively affected mortar strength [51]. Studies also highlighted the importance of controlled laboratory conditions for reliable testing, as opposed to outdoor exposure [52]. Comparative analyses of PCCs containing different polymers, such as SBR, styrene–acrylic ester, and polyacrylic ester, revealed that SBR-based PCCs outperformed others in strength, weight loss, and resistance to chloride ion penetration [53]. Similarly, epoxy-based PCCs exhibited superior strength and durability compared to acrylic-based counterparts [54]. A detailed comparison of SBR and acrylic polymers concluded that SBR-based composites generally had higher bond strength [55]. On the other hand, no significant differences were observed between PCCs and conventional cement mortars in terms of mechanical properties and penetration resistance [56]. Additionally, variations in water-to-cement ratios (0.38 to 0.47) had negligible impact on the adhesion between PCCs and the substrate concrete [57].

Table 1 presents the representative polymer types used for repairing deteriorated concrete structures [44,45,46,47,48,49,50,53,54,55]. Traditional repair materials, such as thermoset resins and epoxies, offer notable advantages but face significant limitations in durability and recyclability. Their susceptibility to extreme thermal or chemical conditions, coupled with brittleness under repeated stress, often leads to premature failure, particularly in high-stress applications or environments subjected to dynamic loads. Additionally, micro-damages and cracks in these materials tend to propagate over time due to the absence of intrinsic self-healing properties, further undermining structural integrity. These challenges are exacerbated in harsh environments, such as coastal regions, freezing climates, or high-temperature zones, where factors like saltwater corrosion, freeze–thaw cycles, and thermal expansion accelerate degradation. Prolonged repair operations intensify these issues by causing service disruptions that impact transportation, commerce, and daily life. Addressing these limitations requires innovative solutions, and emerging materials like epoxy vitrimers show promising potential for faster, more durable, and reliable repairs.

## 3. Reshaping and Recycling of Epoxy-Based Vitrimers

**Figure 3:**
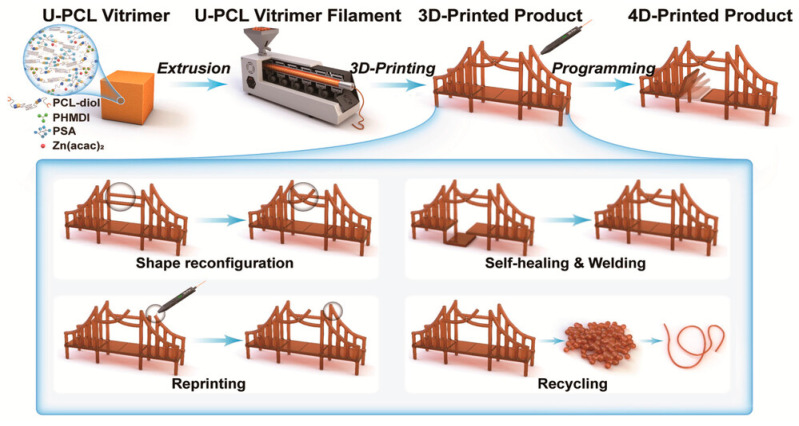
Schematic representation of a 4D printing process of a U-PCL vitrimer via filament extrusion and handheld FDM-based 3D printing, and its multiple functions including shape reconfiguration, self-healing, repair by welding and reprinting, and recycling [64]. Copyright 2021 Wiley.

Transesterification, also known as alcoholysis, is a chemical reaction between oil or fat and alcohol. This process, carried out in the presence of a catalyst—such as an acid, alkali, or enzyme—produces esters and glycerol. This equilibrium-based reaction is essential in polymer chemistry, particularly for creating materials with dynamic properties. Vitrimers, a class of polymers capable of rearranging their internal structure, utilize this reaction to exhibit unique temperature-dependent behaviors. When the temperature is below their topology freezing transition temperature (*T_v_*), vitrimers behave like traditional thermosets, demonstrating excellent thermal and mechanical stability. However, above *T_v_*, vitrimers transform into viscoelastic fluids through topological rearrangements driven by transesterification [58,59]. Although vitrimers offer many promising properties, their limitation is mainly based on the insufficient reactivity of their transesterification-based networks, which hinders efficient reprocessing. Strategies to address this limitation include using catalysts, increasing the concentration of hydroxyl groups, and reducing crosslink density [32,60,61,62,63]. Figure 3 presents a schematic overview of the 4D printing process for a dynamic hindered urea-linked, semicrystalline poly(ε-caprolactone) (PCL) vitrimer, illustrating how both filament extrusion and handheld FDM-based 3D printing enable shape reconfiguration, self-healing, repair via welding and reprinting, and eventual recycling [64].

Epoxy-based vitrimers typically utilize dynamic covalent bonds—often formed via transesterification reactions—as the underlying mechanism for their recyclability [65,66,67,68]. At elevated temperatures, these dynamic bonds undergo reversible exchange, enabling network rearrangements without compromising the material’s structural integrity. Upon cooling, the bonds re-form, allowing the material to retain its shape and mechanical properties. Catalysts can play a crucial role in transesterification reactions and can be acidic, basic, amine-based, organometallic, or even enzyme-based [14,28,31]. Zinc salts, triphenylphosphine, triazobicyclodecene, and tertiary amines are commonly known examples of effective catalysts for vitrimers [65,66,67,68]. Table 2 below summarizes the type of catalyst for transesterification in vitrimers [14,28,31,65,66,67,68,69].

Epoxy-based vitrimers are among the most extensively studied vitrimer systems [70]. These systems rely on transesterification reactions between esters and beta-hydroxyl groups generated during the reaction of epoxy precursors with acids or anhydrides [71]. As mentioned previously, catalysts are essential for accelerating these reactions, allowing the material to relax stress and undergo topological rearrangements in the crosslinked network without altering the overall crosslink density [72]. The chemical structure of the curing agent and the epoxide equivalent of the epoxy oligomers are two key factors in determining the network structure of epoxy-based vitrimers. To balance desirable thermo-mechanical properties with sufficient free hydroxyl groups for reactivity, careful adjustment of the epoxy-to-carboxyl ratio is crucial [73,74,75,76].

**Figure 4:**
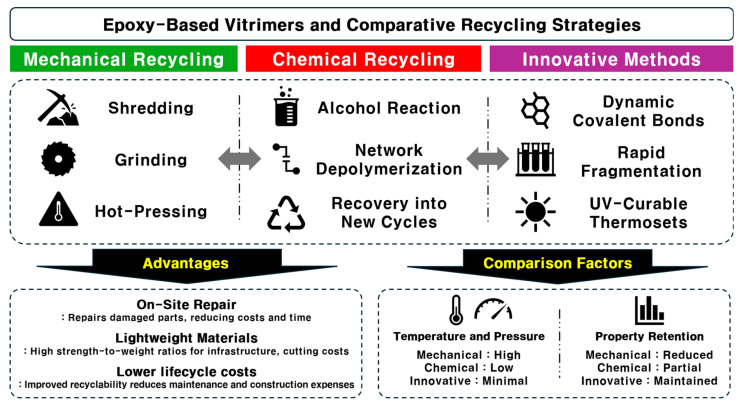
Recycling strategies for thermoset composites in sustainable construction.

The recycling processes for epoxy-based vitrimers can be broadly categorized into thermal reprocessing, chemical recycling, mechanical recycling, and solvent-based recycling, each leveraging the unique dynamic covalent chemistry inherent to vitrimers [77,78,79,80]. Thermal reprocessing involves heating the vitrimer above its glass transition temperature to activate reversible bond exchange reactions, allowing the material to be reshaped, repaired, or reformed without significant degradation of its mechanical properties, thereby extending its lifecycle. Chemical recycling focuses on depolymerizing the polymer network into monomeric or oligomeric components through specific chemical reactions, such as introducing catalysts or reagents that selectively cleave dynamic bonds, enabling the recovery and repolymerization of original chemical constituents and reducing reliance on virgin resources. Mechanical recycling entails physically processing vitrimer waste by grinding it into smaller particles for reintroduction into the manufacturing process, which, although it may not fully exploit dynamic bond exchange capabilities, offers a straightforward and cost-effective method for material reuse, especially when combined with thermal or chemical treatments to restore network integrity. Figure 4 exhibits three representative recycling approach for thermoset composites. With increasing emphasis on sustainability, recycling thermoset composites has become critical. Mechanical recycling involves shredding and grinding composites into smaller pieces and hot-pressing them into new forms. However, this method requires high temperatures and pressures, often resulting in degraded mechanical properties. For instance, fiber-reinforced polymeric composites (FRPCs) made with reversible disulfide bonds have been reported to be fully recyclable while maintaining mechanical integrity [80]. However, this process may not be cost-effective and could compromise structural integrity, restricting its use to non-structural applications.

Chemical recycling presents a more promising alternative. Chemical recycling of vitrimers involves breaking down their dynamic covalent networks into smaller molecules or monomers, enabling the recovery and reuse of raw materials. This process leverages the reversible bond exchange mechanisms inherent in vitrimers, such as transesterification or imine exchange, to facilitate depolymerization under controlled conditions. Specifically, small-molecule alcohols like ethylene glycol (EG) or ethanol also can be used to break down the polymer network into smaller fragments [81]. After the solvent evaporates, the material is ready for new curing cycles. The effectiveness of this chemical method has been further demonstrated by separating fibers from an epoxy-anhydride matrix using various solutions such as 1,5,7-triazabicyclo [4,4,0]dec-5-ene, *N*-methyl-2-pyrrolidone, and EG [79]. The recovered fibers retained excellent morphology and tensile strength. Additionally, this chemical recycling process has been effective in reclaiming intact metal components from electronic devices like light-emitting diodes. Chemical recycling also shows potential for 3D printing applications. For example, recyclable inks were developed from a mixture of bisphenol A diglycidyl ether and fatty acids, enhanced with Zn(Acac)_2_ and nanoclay, allowing consistent reprocessing across multiple cycles [69].

Another innovative recycling method incorporates exchangeable bonds directly into the thermoset backbone, facilitating easier dissolution into smaller fragments [81]. Disulfide-based epoxy crosslinked with diamines was examined to achieve rapid fragmentation within an hour. Although promising for composite recycling, this approach requires specific chemical formulations [82]. UV-curable thermosets are another promising approach for reprocessable materials. A system involving acrylate monomers, crosslinkers, and zinc-based catalysts enabled repairs and recycling through exchange reactions [83]. This method successfully restored the mechanical properties of damaged parts and allowed mechanical recycling into non-structural components.

Recycling strategies for vitrimers and other thermosets are advancing. Mechanical, chemical, and hybrid methods offer varying levels of success. While each recycling method has limitations, advancements in material design and processing are paving the way for more sustainable and versatile recycling options for industrial applications. The potential of reprocessable and recyclable vitrimers extends to civil engineering, particularly in the construction and maintenance of infrastructure. As mentioned previously, traditional construction materials like concrete and conventional thermosets face challenges related to sustainability, lifecycle costs, and waste management. Vitrimers, with their dynamic covalent bonding and capacity for topological rearrangement, offer innovative solutions to these challenges. For example, vitrimer-based materials can be repaired in situ, reducing the need for full replacements and significantly cutting maintenance costs and downtime. This feature is particularly valuable in remote or difficult-to-access areas, where structural replacements are costly and logistically challenging. In addition to their repairability, vitrimers hold a strong potential for designing lightweight, high-performance materials suitable for structural applications. By combining vitrimer recyclability with the excellent mechanical properties of fiber reinforced composites (FRCs), it is possible to create materials with high strength-to-weight ratios. Such materials are ideal for large-scale infrastructure projects like bridges, tunnels, and wind turbine blades, where reducing weight without sacrificing strength can lead to significant cost savings in transportation, installation, and overall structural performance. Thus, vitrimers’ reprocessable nature, adaptability, and sustainability make them a promising innovation for transforming the construction and maintenance of civil infrastructure.

## 4. Self-Healing of Epoxy-Based Vitrimers

**Figure 5:**
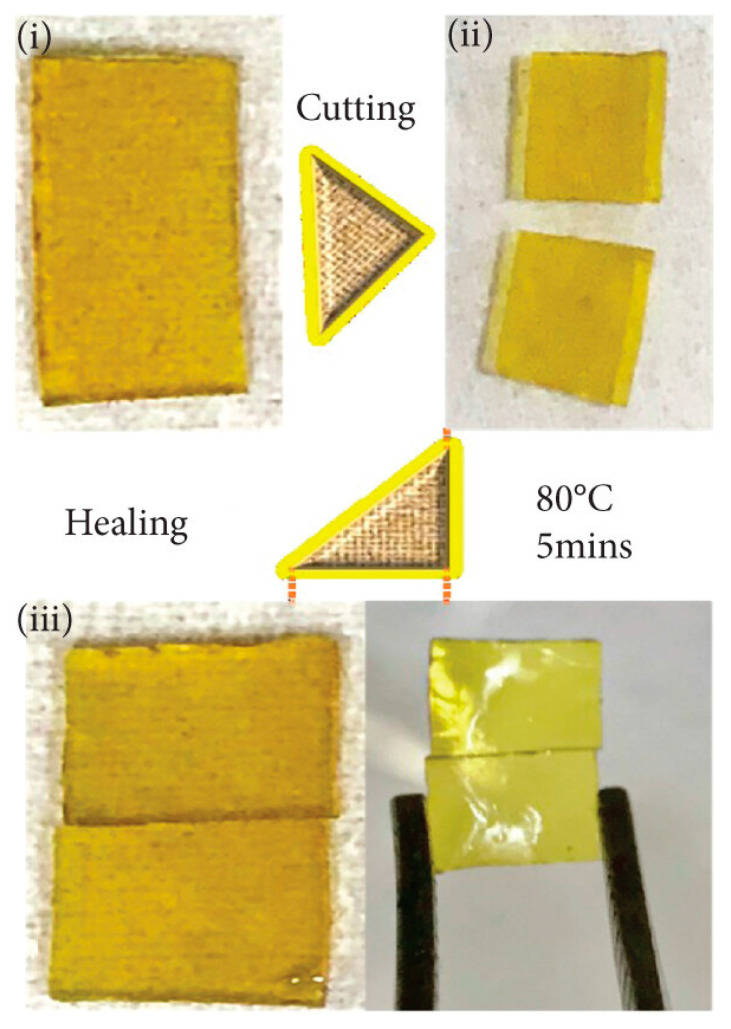
Self-healing of epoxy vitrimer: (**i**) prinstine EP-p, (**ii**) cut into two pieces, and (**iii**) rejoined [88]. Copyright 2021 Wiley.

Self-healing in vitrimers is a groundbreaking development enabled by dynamic covalent bonding networks that allow autonomous repair of damage through bond exchange reactions under specific stimuli [84]. These materials combine the stability of thermosets with the reprocessability of thermoplastics, making them uniquely suitable for structural applications [13,19,26]. The self-healing process involves viscoelastic flow and the activation of dynamic bonds triggered by stimuli such as heat, light, or chemical catalysts. Upon damage, such as crack formation, disrupted CANs are restored by bond dissociation and reassociation [85]. Heat above the vitrimer’s glass transition temperature typically activates these dynamic bonds, enabling viscoelastic flow to bring damaged surfaces into contact and reform bonds, often restoring mechanical properties to levels comparable to the original state. Some systems utilize catalysts or photo-responsive groups to lower activation energy or trigger healing via ultraviolet (UV) light, broadening operational conditions [86,87]. Figure 5 illustrates the self-healing process of an epoxy vitrimer, transitioning from a pristine EP-p (i) to two separate pieces (ii), and eventually rejoining (iii), thus demonstrating its robust self-repair capabilities [88].

Epoxy-based vitrimers enhance this capability by incorporating dynamic covalent chemistries such as transesterification, disulfide exchange, or imine exchange [89,90]. Disulfide linkages enable healing at lower temperatures, while imine-based systems provide additional pathways for chemically demanding environments. These mechanisms ensure not only self-healing but also excellent mechanical properties, making epoxy-based vitrimers particularly attractive for load-bearing applications. Performance is evaluated through metrics like healing efficiency, healing time, and operational range. Healing efficiency measures the recovery of mechanical properties, healing time reflects the duration needed for repair, and operational range defines the conditions under which healing is effective. Table 3 highlights how vitrimers uniquely combine high strength, durability, and reprocessability, making them versatile and sustainable compared to other self-healing materials [91,92,93,94,95,96,97,98,99,100,101,102,103,104,105]. When compared to other self-healing materials, vitrimers stand out for their intrinsic ability to heal repeatedly without external agents. Polymers with encapsulated healing agents, while effective for rapid autonomous repair, are limited to being single-use and may not restore the full strength of the original material [91,92]. Supramolecular polymers heal through non-covalent interactions, such as hydrogen bonding or metal-ligand coordination, which enable low-energy healing but lack the mechanical strength and durability required for structural applications [93,94]. Thermoplastic elastomers heal through thermal softening, similar to vitrimers, but their limited rigidity makes them unsuitable for high-performance uses [95,96,97,98]. Shape memory polymers rely on programmed shape recovery to close cracks, yet their healing does not restore molecular-level integrity, leading to reduced mechanical properties after repeated cycles [99,100]. Hydrogels, while excellent for wet conditions and biomedical uses, lack the strength and environmental resistance needed for industrial or structural applications [101,102,103].

In contrast, vitrimers, particularly epoxy-based variants, offer repeated self-healing through dynamic covalent bonds, superior mechanical strength, and durability under harsh conditions [104,105]. They combine thermoset-like rigidity with thermoplastic-like reprocessability, making them ideal for structural, aerospace, and automotive applications. They outperform other self-healing materials in terms of recyclability, as they can be reprocessed multiple times without degrading mechanical properties. This feature also aligns with sustainability goals by reducing material waste and extending the lifespan of components. The key advantages of vitrimers include their ability to repeatedly heal damage, restore molecular-level integrity, and maintain high mechanical strength, making them suitable for diverse and demanding applications. They also resist environmental degradation better than hydrogels or thermoplastic elastomers, making them versatile for use in extreme conditions. While challenges such as high activation temperatures, scalability, and balancing healing capability with mechanical strength remain, ongoing research focuses on improving these aspects. For instance, integrating nanomaterials like graphene can enhance mechanical and thermal properties, and developing catalysts or multi-stimuli responsive systems can reduce activation requirements.

Overall, the self-healing properties of vitrimers, especially epoxy-based systems, position them as a transformative solution in advanced materials. Their ability to autonomously repair damage under diverse stimuli, coupled with their exceptional structural integrity, addresses critical challenges in high-performance applications. When compared to other self-healing materials, vitrimers clearly demonstrate superior longevity, reusability, and environmental sustainability. With continued innovation, they hold promise for revolutionizing fields ranging from aerospace to civil engineering, offering both performance and sustainability in equal measure.

## 5. Limitations and Future Works for the Application of Vitrimers in Civil Infrastructures

**Figure 6:**
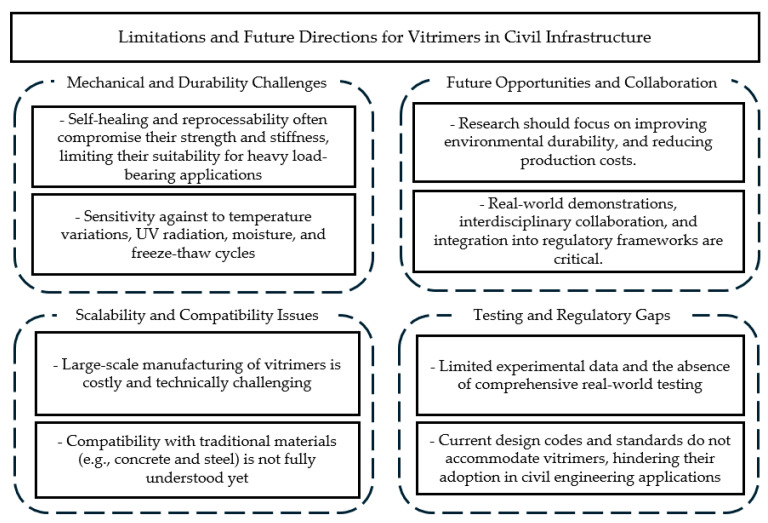
Limitations and future prospects for the application of vitrimers in civil infrastructure.

Vitrimers are unique in that they contain dynamic covalent bonds—often enabled by transesterification—capable of breaking and reforming under specific conditions. At elevated temperatures or in the presence of catalysts, these bonds undergo reversible exchange, allowing the polymer network to reorganize without losing its overall integrity. This mechanism underpins the material’s self-healing ability: when damage such as microcracks occur, local heating causes bond exchange, effectively “filling in” the damaged region and restoring structural cohesion. The same principle facilitates recycling, as thermally activated bond rearrangements allow a vitrimer to be softened, reshaped, and remolded multiple times. These repeated reprocessing cycles can be achieved through methods like hot-pressing or extrusion, during which the polymer network becomes sufficiently mobile for reorganization, yet retains solid, robust properties upon cooling. However, their application in civil engineering structures is not without limitations, and considerable future work is needed to enlarge their full potential. One primary limitation lies in the mechanical properties of vitrimers. While their dynamic covalent bonding enables reprocessing and self-healing, these features often come at the expense of strength and stiffness, making them less suitable for heavy load-bearing applications [106,107]. Improving the balance between reprocessability and structural performance remains a challenge. Additionally, thermal and environmental stability pose significant concerns. Vitrimers are sensitive to temperature variations, which could limit their functionality in high-temperature environments or during fire scenarios. Prolonged exposure to environmental factors like UV radiation, moisture, and freeze–thaw cycles further complicates their long-term use. Figure 6 summarizes the limitations and future prospects for the application of vitrimers in civil infrastructure.

In construction, vitrimers can be employed as coatings, adhesives, and sealants that can heal microcracks and other forms of wear arising from structural stress or environmental factors such as freeze–thaw cycles. For instance, a self-healing coating on a concrete tunnel wall could mitigate water penetration and chloride ingress, reducing the corrosion of embedded steel reinforcements. Likewise, vitrimers used in expansion joints or as structural adhesives can be heated to reverse or reform bonds, facilitating disassembly and reassembly during renovations or retrofits. This reconfigurability also extends to composite reinforcement systems, where fiber-reinforced vitrimer wraps can be removed or repurposed if the structural requirements change over time. Overall, the adaptability and damage-tolerant nature of vitrimers make them promising for enhancing durability, extendability, and sustainability in large-scale construction projects.

From a practical perspective, scalability and manufacturing challenges hinder the widespread adoption of vitrimers [72,108,109]. The production of these materials often requires precise temperature control and specific catalysts, making large-scale manufacturing costly and technically demanding. Moreover, their compatibility with traditional construction materials such as concrete and steel is not fully understood. Issues such as adhesion, thermal expansion mismatches, and chemical interactions could negatively influence on structural integrity. Furthermore, limited experimental data and lack of comprehensive testing under real-world conditions create uncertainties regarding their long-term performance. Current design codes and standards do not accommodate the use of vitrimers, adding another layer of complexity to their adoption in structural applications.

Most studies indicate that the mechanical and thermal properties of vitrimers remain relatively stable across multiple recycling cycles [9,110,111]. For example, tensile strength, elastic modulus, and glass transition temperature often exhibit minimal shifts, typically within a margin of 10–20% loss compared to the original material. Small decreases in these properties, when observed, can stem from thermal oxidative degradation at high temperatures, incomplete bond exchange, or minor changes in cross-link density if the network architecture is altered. The use of appropriate catalysts and carefully controlled processing conditions can further mitigate property loss, enabling repeated recycling without significant performance deterioration. As such, while some aging or degradation may be inevitable with multiple reprocessings, vitrimeric materials generally maintain much of their initial structural integrity and remain suitable for demanding construction applications over an extended service life. To address mentioned limitations above, future work should focus on enhancing the mechanical properties of vitrimers through molecular engineering, composite reinforcements, and hybrid material systems. The incorporation of nanomaterials like graphene or carbon nanotubes could offer significant improvements in strength and toughness. Efforts to improve thermal and environmental durability through advanced vitrimer chemistries are equally important. For example, formulations that retain dynamic bonding properties under extreme conditions could expand their usability.

Large-scale manufacturing techniques, such as additive manufacturing or automated lay-up processes, require further development to reduce production costs and improve material consistency. Testing programs, including accelerated aging, fatigue analysis, and failure testing under diverse loading conditions, will provide critical data for validating vitrimer performance. Predictive computational models simulating vitrimer behavior under structural and environmental stresses will aid in optimizing their design. Field demonstrations and pilot projects will be invaluable in proving the practical application of vitrimers. Real-world case studies in areas such as retrofitting, modular construction, or self-healing infrastructure can provide insights into their performance and cost-effectiveness [112,113,114]. Interdisciplinary collaboration between materials scientists, chemists, and structural engineers is essential for tailoring vitrimer properties to specific applications and accelerating the transition from laboratory research to real-world implementation.

Vitrimers have demonstrated strong potential in the construction sector, offering a range of applications that capitalize on their dynamic covalent bonding and self-healing properties. For instance, when used as self-healing coatings on concrete, vitrimers can repair microcracks triggered by mechanical stress or freeze–thaw cycles, thus extending the lifespan of tunnels, bridges, and other infrastructure. These materials also function as robust but reprocessable adhesives in retrofitting projects, where heated vitrimers can be disassembled without damaging key structural elements. Moreover, vitrimers can serve as waterproofing membranes and sealants capable of resisting chloride penetration while healing damage caused by temperature fluctuations or mechanical wear. Beyond protective coatings, vitrimer-based formwork simplifies demolding and potentially allows for shape-memory functions, enabling adjustable formwork geometries. When reinforced with fibers (e.g., carbon or glass), vitrimers can be deployed as load-bearing composite wraps that can be thermally reprocessed and recycled, reducing end-of-life waste. In addition, expansion joints and sealants formulated with vitrimer resins can heal tears under localized heating, enhancing performance in high-stress junctions. Finally, vitrimer-containing modular components can be reconfigured or recycled, promoting a circular economy in large-scale construction projects and reducing overall material consumption.

In previous studies, epoxy vitrimers have primarily been examined under controlled laboratory conditions, providing valuable insights into their mechanical properties, recyclability, and environmental performance. However, these conditions do not fully capture the complexities of real-world industrial applications. Future research should address this gap by collaborating with industry partners and other stakeholders to conduct pilot-scale demonstrations, refine formulations for large-scale production, and evaluate performance in operational settings. Additionally, comprehensive life-cycle assessments and strict adherence to environmental regulations will be crucial for responsible commercialization. Such collaborations will enable epoxy vitrimers to transition from laboratory research to practical, widespread use, thereby advancing sustainable development objectives. Sustainability analysis of vitrimers, including lifecycle assessments quantifying their recyclability and environmental impact, will strengthen their position as a sustainable alternative to traditional materials. Integrating vitrimers into regulatory frameworks and building codes is crucial for their widespread acceptance. Collaboration with industry stakeholders, regulatory bodies, and researchers will help establish performance metrics and safety standards. Innovative applications, such as self-healing roadways, adaptive building facades, or disaster-resilient structures, should be explored to expand the scope of vitrimer use in civil engineering. These advancements will not only demonstrate the versatility of vitrimers but also address critical challenges faced by modern infrastructure. While vitrimers hold immense promise for transforming structural engineering, significant challenges related to their mechanical properties, environmental durability, scalability, and regulatory acceptance need to be addressed.

## 6. Conclusions

This review systematically examines the dynamic recycling processes, self-healing mechanisms, and emerging applications of epoxy-based vitrimers in civil engineering. Based on a comprehensive analysis and comparison, the following key conclusions are drawn:

1. Unlike traditional thermosets, epoxy vitrimers can undergo multiple cycles of reprocessing and repair without compromising their mechanical integrity. This recyclability significantly reduces material waste and lifecycle costs, thereby supporting a circular economy and minimizing environmental impact.
2. Epoxy-based vitrimers utilize dynamic covalent bonds, such as transesterification, to autonomously repair microcracks and structural defects. This self-healing ability extends the lifespan of infrastructure exposed to harsh environmental conditions, including freeze–thaw cycles, UV radiation, and chemical exposure, thereby reducing maintenance requirements and ensuring structural reliability.
3. The incorporation of bio-based feedstocks, such as lignin and rosin derivatives, enhances the environmental compatibility of vitrimers. By providing eco-friendly alternatives to conventional materials, these advancements align with global sustainability goals and promote greener material solutions.
4. Vitrimers demonstrate strong potential in various applications, including concrete surface coatings, adhesives, and sealants. These materials can autonomously repair surface damage, prevent crack propagation, and enhance the durability of structural joints, particularly in modular construction and high-stress environments.

In summary, epoxy-based vitrimers exhibit transformative potential in addressing critical challenges within civil infrastructure. Their unique combination of self-healing and recyclable properties offers sustainable, resilient, and cost-effective solutions. These findings highlight the capacity of vitrimers to revolutionize infrastructure design and maintenance, thereby aligning with modern sustainability and performance objectives.

## Figures and Tables

**Table 1:** Representative polymer types used for repairing deteriorated concrete structures [44,45,46,47,48,49,50,53,54,55].

Polymer Type	Chemical Formula	Strengths	Weakness	Refs.
Styrene Butyl Acrylate	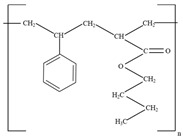	-Improved cracking resistance compared to other polymer-modified self-leveling mortars	-As the dosage increases, the tensile strength of the mortar can be reduced	[44]
Epoxy resin	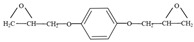	-Excellent compatibility with cement-based materials -Strong adhesion under thermal stress caused different thermal expansion coefficients (CTEs)	-Exceeding 10% dosage might disrupts hydration and polymerization processes, leading to a reduction in mechanical properties	[45,46,47,48]
Styrene Butadien Rubber (SBR)	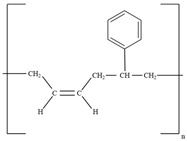	-Significantly reduced water absorption of concrete (by 40% to 49%). -Improved freeze–thaw resistance	-Excessive incorporation can reduce the compressive strength of concrete	[49,50]
Styrene acrylic polymer	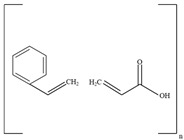	-Excellent plasticizing effect and delayed setting behavior in cement mortar	-Weaker in terms of mechanical strength compared to SBR	[53]
Polyacrylic ester	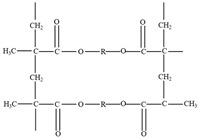	-Excellent resistance against to carbonation and sulfate erosion in cementitious composites	-Low compressive strength compared to the control with no additives.	
Acrylic polymer	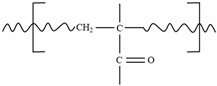	-Resistance against to compression and wear, lower permeability, and improved bond strength	-Exhibited low shrinkage resistance when dry curing was done	[54,55]

**Table 2:** Overview of catalysts for transesterification in vitrimers [14,28,31,65,66,67,68,69].

Catalyst Type	Examples	Chemical Formular	Role	Refs.
Acidic Catalysts	Zinc salts	Zn(OAc)₂ or ZnCl₂	Accelerates transesterification reaction	[65,66,67,68]
Basic Catalysts	Triphenylphosphine (TPP) Triazabicyclodecene (TBD)	P(C₆H₅)₃ C₆H₁₂N₄	Facilitates network rearrangement	[65,66,67,68]
Amine-Based Catalysts	Tertiary amines	N(R₁)(R₂)(R₃)	Activates dynamic bond exchange reactions	[31]
Organometallic Catalysts	Zinc acetylacetonate	Zn(C₅H₇O₂)₂	Promotes bond exchange reactions and enables network reconfiguration	[69]
Enzyme-Based Catalysts	Lipase	Protein-based enzyme	Acts as a nature-friendly catalyst in ester exchange reactions	[14,28]
Mixed Catalysts	Zinc salts + TPP	Zn(OAc)₂ + P(C₆H₅)₃	Synergistic effects in accelerating transesterification reactions	[65,68]

**Table 3:** Comparisons of various self-healing materials and its applications [91,92,93,94,95,96,97,98,99,100,101,102,103,104,105].

Material Type	Mechanism	Strengths	Conclusions	Best Fit	Refs.
Polymers with Encapsulated Healing Agents	Encapsulated agents released at the damage site	Rapid, autonomous repair	Single-use; may not restore full strength of the original material	Temporary repairs	[91,92]
Supramolecular Polymers	Non-covalent interactions (e.g., hydrogen bonding, metal-ligand coordination)	Low-energy; reversible bonding	Lack mechanical strength and durability for structural uses	Flexible, non-load uses	[93,94]
Thermoplastic Elastomers	Thermal softening	Repeated self-healing capability	Limited rigidity; unsuitable for high-performance, structural applications	Minor structural elements	[95,96,97,98]
Shape Memory Polymers	Programmed shape recovery	Effective for closing cracks	Healing does not restore molecular-level integrity; reduced mechanical properties with cycles	Crack sealing	[99,100]
Hydrogels	Water retention and swelling	Excellent for wet environments and biomedical applications	Lack strength and environmental resistance for industrial uses	Biomedical, wet environments	[101,102,103]
Vitrimers	Dynamic covalent bonds	Repeated self-healing; superior mechanical strength and durability; thermoset-like rigidity with thermoplastic-like reprocessability; recyclable	Requires specific conditions for bond rearrangement	Structural applications	[104,105]

## Data Availability

Not applicable.
